# Supplementary material for: Human–Animal Bond Generated in a Brief Animal-Assisted Therapy Intervention in Adolescents with Mental Health Disorders
Source: Animals (Basel). 2023 Jan 20;13(3):358. doi: 10.3390/ani13030358 (PMC9913319; doi:10.3390/ani13030358)
Supplement: Supplementary file 1 [file animals-13-00358-s001.zip › animals-2139846-supplementary.pdf]

SUPLEMENTARY MATERIAL

1. **File S1: Description of dynamics**

Dynamic 1: AGILITY CIRCUIT

Plan and set up a circuit as an agility track with hoops, cones, and poles. There were parts of the circuit that the participant and dog could go through in tandem, and parts where the participant had to get the dog to go through without a leash (they could use food to help the dog).

Dynamic 2: WE ARE TRAINERS

Using methodologies for working respectfully with animals, the adolescent(s) learned how to teach a dog to perform behaviors, and by the same token, learned non-violent learning methodologies based on communication and cooperation.

Dynamic 3: DOG TOYS

They made cognitive toys for dogs and then were able to play and interact with the constructed toys and the dog; they were taught respectful and fun ways to play in which goals were achieved based on cooperation.

Different materials were provided and before starting they thought/planned and discussed: what they could make and how they could play with the dog.

Dynamic 4: WHICH BOX DO I GET?

We placed some laminated sheets on the floor; each sheet had instructions to perform. The dog would be given a rope-chew that they had to throw and then follow the instructions printed on the sheet where the rope fell.

Example of instructions:

- Act out a movie using mime and have peers guess what the movie is
- Draw a picture while blindfolded (the group decides what to draw)
- Ask the dog to perform a skill
- Give a compliment to each participant
- Explain a current news item

Dynamic 5: TRAFFIC LIGHT

In this activity, real situations were presented where they had to interpret which reaction would be most appropriate. These were situations that we can classify using the traffic light technique: green (desirable), yellow (improvable), and red (inappropriate).

Situations that favored group cohesion, empathy, and interpersonal relationships were presented. A green hoop, a yellow hoop, and a red hoop were placed on the floor. The participant had to walk with the dog and stop at the hoop in the color he/she thought appropriate.

It facilitated discussion about each situation among the participants, about whether they all felt the same way, how it could be improved, what to avoid and why, etc.

Dynamic 6: CANINE EMOTIONS

In this dynamic we talked about different dog emotions and how we can interpret what dogs feel. We explained dog body language and signs of calm, and compared with our body language.

It was always compared to our feelings and the situations that trigger them.

It was decided to carry out an intervention of only 2 sessions due to participants' short stays at the center, as these are acute units and a longer intervention would entail the loss of many participants.

2. Table S1: "Center for the Study of Animal Wellness Pet Bonding Scale (CSAWPBS)" general results

CSAWPBS	ALL *[N=114]*	
Human-dog Bond	56.4 (18.1)	114
1. The dog visitor likes me.	1.71 (0.81)	114
2. I talk to the dog visitor.	1.75 (1.10)	114
3. I confide in the dog visitor.	1.43 (0.82)	114
4. The dog visitor understands what I say.	1.91 (1.11)	113
5. The dog visitor knows when I feel bad.	2.59 (1.28)	114
6. The dog visitor is always glad to see me.	2.18 (1.16)	114
7. The dog visitor prefers me to others.	3.95 (1.12)	114
8. The dog visitor has become my friend.	2.01 (1.23)	114
9. I look forward to getting up in the morning on days	2.03 (1.30)	113

when I will see the dog visitor.		
10. I tell others about the dog visitor.	2.30 (1.39)	114
11. The dog visitor knows when I feel happy	2.30 (1.25)	114
12. I would like to have the dog visitor come to my home.	2.06 (1.45)	114
13. I will remember the dog visitor after my program.	1.32 (0.79)	114
14. The dog visitor makes walking easier.	2.62 (1.45)	114
15. The dog visitor tries to comfort me.	1.92 (1.11)	113
16. The dog visits make me feel better.	1.62 (0.92)	113
17. The dog visits are (not) boring.	1.62 (1.02)	114
18. I feel attached to the dog visitor.	2.43 (1.33)	113
19. The dog visits give me energy.	1.93 (1.11)	114
20. I miss the dog visitor between visits.	2.34 (1.48)	114
21. The dog doesn't judge me.	1.30 (0.87)	114
22. I look forward to the dog visits.	1.74 (1.08)	114
23. The dog visits make me feel happy.	1.61 (0.89)	114
24. The dog accepts me just the way I am.	1.26 (0.62)	114
25. I make the dog feel better.	2.35 (1.11)	112
26. I make the dog feel happy.	2.19 (1.09)	112
27. The dog takes my mind off my troubles.	1.96 (1.32)	112
2 The dog helps me feel secure.	2.00 (1.29)	112

Total score range: from 28 (always true) to 140 (never true). Individual item range: from 1 (always true) to 5 (never true).

3. **Supplementary Table S2: "Center for the Study of Animal Wellness Pet Bonding Scale (CSAWPBS)" participant-dog bond according to pet ownership results**

CSAWPBS	No (N=89)	Yes (N=25)	T-value (Yes vs No)	p-value
1. The dog visitor likes me.	1.73 (0.82)	1.64 (0.76)	-0.517	0.608
2. I talk to the dog visitor.	1.81 (1.17)	1.52 (0.77)	-1.463	0.149
3. I confide in the dog visitor.	1.44 (0.84)	1.40 (0.76)	-0.216	0.83
4. The dog visitor understands what I say.	1.91 (1.09)	1.92 (1.18)	0.025	0.981
5. The dog visitor knows when I feel bad.	2.64 (1.25)	2.40 (1.38)	-0.783	0.439
6. The dog visitor is always glad to see me.	2.20 (1.17)	2.08 (1.15)	-0.467	0.643
7. The dog visitor prefers me to others.	4.03 (1.03)	3.64 (1.38)	-1.326	0.194
8. The dog visitor has become my friend.	2.01 (1.27)	2.00 (1.08)	-0.044	0.965
9. I look forward to getting up in the morning on days when I will see the dog visitor.	2.07 (1.33)	1.88 (1.20)	-0.675	0.504
10. I tell others about the dog visitor.	2.42 (1.44)	1.88 (1.13)	-1.966	0.055
11. The dog visitor knows when I feel happy	2.31 (1.25)	2.24 (1.30)	-0.256	0.8
12. I would like to have the dog visitor come to my home.	2.19 (1.49)	1.60 (1.19)	-2.068	0.044
13. I will remember the dog visitor after my program.	1.35 (0.83)	1.24 (0.66)	-0.681	0.499
14. The dog visitor makes walking easier.	2.62 (1.51)	2.64 (1.25)	0.074	0.941
15. The dog visitor tries to comfort me.	1.85 (1.13)	1.72 (1.06)	-0.542	0.591
16. The dog visits make me feel better.	1.68 (0.95)	1.40 (0.76)	-1.536	0.131
17. The dog visits are (not) boring.	1.61 (1.02)	1.68 (1.03)	0.315	0.754
18. I feel attached to the dog visitor.	2.39 (1.34)	2.58 (1.32)	0.626	0.535
19. The dog visits give me energy.	1.92 (1.09)	1.96 (1.21)	0.144	0.886
20. I miss the dog visitor between visits.	2.43 (1.54)	2.04 (1.24)	-1.304	0.199
21. The dog doesn't judge me.	1.29 (0.84)	1.32 (0.99)	0.129	0.899
22. I look forward to the dog visits.	1.82 (1.13)	1.44 (0.82)	-1.869	0.067
23. The dog visits make me feel happy.	1.66 (0.95)	1.40 (0.58)	-1.714	0.091
24. The dog accepts me just the way I am.	1.31 (0.68)	1.08 (0.28)	-2.571	0.012
25. I make the dog feel better.	2.35 (1.18)	2.33 (0.82)	-0.091	0.928
26. I make the dog feel happy.	2.16 (1.13)	2.29 (0.95)	0.578	0.566
27. The dog takes my mind off my troubles.	2.07 (1.38)	1.54 (0.98)	-2.125	0.038
28. The dog helps me feel secure.	2.06 (1.37)	1.79 (0.93)	-1.107	0.273

Total score range: from 28 (always true) to 140 (never true). Individual item range: from 1 (always true) to 5 (never true).

Supplementary Table S3: "Center for the Study of Animal Wellness Pet Bonding Scale (CSAWPBS)" participant-dog bond according to diagnosis results

CSAWPBS	Depression				Psychosis				Behavioral disorder				Eating disorder			
	No (N=65)	Yes (N=49)	T-value (Yes vs No)	*p-value*	No (N=109)	Yes (N=5)	T-value (Yes vs No)	*p-value*	No (N=101)	Yes (N=13)	T-value (Yes vs No)	*p-value*	No (N=74)	Yes (N=40)	T-value (Yes vs No)	*p-value*
1. The dog visitor likes me.	1.65 (0.76)	1.80 (0.87)	0.964	0.338	1.72 (0.82)	1.40 (0.55)	-1.263	0.264	1.68 (0.80)	1.92 (0.86)	0.952	0.356	1.73 (0.83)	1.68 (0.76)	-0.354	0.7525
2. I talk to the dog visitor.	1.74 (1.12)	1.76 (1.07)	0.081	0.936	1.75 (1.12)	1.60 (0.55)	-0.57	0.591	1.76 (1.09)	1.62 (1.19)	-0.422	0.679	1.66 (1.02)	1.90 (1.22)	1.052	0.296
3. I confide in the dog visitor.	1.38 (0.84)	1.49 (0.79)	0.682	0.497	1.43 (0.83)	1.40 (0.55)	-0.121	0.908	1.47 (0.86)	1.15 (0.38)	-2.316	0.027	1.34 (0.63)	1.60 (1.08)	1.411	0.164
4. The dog visitor understands what I say.	1.95 (1.15)	1.85 (1.05)	-0.478	0.633	1.93 (1.12)	1.60 (0.55)	-1.217	0.272	1.90 (1.08)	2.00 (1.35)	0.256	0.802	1.89 (1.15)	1.95 (1.04)	0.281	0.779
5. The dog visitor knows when I feel bad.	2.62 (1.38)	2.55 (1.16)	-0.271	0.787	2.61 (1.28)	2.20 (1.30)	-0.681	0.531	2.67 (1.25)	1.92 (1.38)	-1.862	0.083	2.38 (1.26)	2.98 (1.25)	2.427	0.017
6. The dog visitor is always glad to see me.	2.15 (1.16)	2.20 (1.17)	0.227	0.821	2.17 (1.17)	2.40 (1.14)	0.45	0.674	2.25 (1.19)	1.62 (0.77)	-2.596	0.017	2.07 (1.15)	2.38 (1.17)	1.347	0.182
7. The dog visitor prefers me to others.	3.92 (1.12)	3.98 (1.13)	0.266	0.791	3.94 (1.13)	4.20 (0.84)	0.678	0.53	4.02 (1.10)	3.38 (1.12)	-1.926	0.073	3.85 (1.15)	4.12 (1.04)	1.287	0.201
8. The dog visitor has become my friend.	2.06 (1.18)	1.94 (1.30)	-0.519	0.605	2.02 (1.23)	1.80 (1.30)	-0.367	0.731	2.02 (1.23)	1.92 (1.26)	-0.262	0.797	1.96 (1.30)	2.10 (1.10)	0.609	0.544
9. I look forward to getting up in the morning on days when I will see the dog visitor.	1.94 (1.11)	2.14 (1.51)	0.799	0.427	2.05 (1.32)	1.60 (0.55)	-1.617	0.154	2.05 (1.31)	1.85 (1.21)	-0.564	0.581	2.01 (1.40)	2.05 (1.10)	0.158	0.875
10. I tell others about the dog visitor.	2.12 (1.33)	2.53 (1.44)	1.543	0.126	2.31 (1.40)	2.00 (1.22)	-0.553	0.607	2.34 (1.38)	2.00 (1.47)	-0.782	0.447	2.26 (1.43)	2.38 (1.31)	0.444	0.658
11. The dog visitor knows when I feel happy	2.22 (1.24)	2.41 (1.27)	0.808	0.421	2.29 (1.26)	2.40 (1.34)	0.174	0.87	2.35 (1.25)	1.92 (1.26)	-1.145	0.27	2.22 (1.25)	2.45 (1.26)	0.947	0.346
12. I would like to have the dog visitor come to my home.	1.97 (1.40)	2.18 (1.51)	0.774	0.441	2.04 (1.43)	2.60 (1.82)	0.684	0.53	2.07 (1.44)	2.00 (1.58)	-0.15	0.883	2.07 (1.50)	2.05 (1.36)	-0.063	0.95
13. I will remember the dog visitor after my program.	1.37 (0.84)	1.27 (0.73)	-0.705	0.482	1.32 (0.80)	1.40 (0.55)	0.307	0.771	1.34 (0.79)	1.23 (0.83)	-0.434	0.67	1.26 (0.70)	1.45 (0.93)	1.146	0.256
14. The dog visitor makes walking easier.	2.54 (1.44)	2.73 (1.48)	0.709	0.48	2.67 (1.46)	1.60 (0.89)	-2.525	0.053	2.61 (1.44)	2.69 (1.60)	0.168	0.869	2.54 (1.44)	2.78 (1.48)	0.815	0.417
15. The dog visitor tries to comfort me.	1.78 (1.04)	1.88 (1.21)	0.416	0.679	1.83 (1.12)	1.60 (0.89)	-0.563	0.6	1.84 (1.11)	1.69 (1.18)	-0.427	0.676	1.79 (1.17)	1.88 (1.02)	0.381	0.704

16. The dog visits make me feel better.	1.55 (0.71)	1.71 (1.14)	0.904	0.369	1.61 (0.93)	1.80 (0.84)	0.491	0.647	1.65 (0.94)	1.38 (0.77)	-1.141	0.27	1.59 (1.01)	1.67 (0.74)	0.434	0.665
17. The dog visits are (not) boring.	1.51 (0.89)	1.78 (1.16)	1.347	0.181	1.65 (1.03)	1.00 (0.00)	-6.598	<0.001	1.64 (1.04)	1.46 (0.78)	-0.761	0.456	1.65 (1.05)	1.57 (0.96)	-0.378	0.706
18. I feel attached to the dog visitor.	2.45 (1.37)	2.41 (1.29)	-0.179	0.858	2.47 (1.34)	1.60 (0.89)	-2.076	0.0094	2.49 (1.33)	2.00 (1.28)	-1.237	0.237	2.27 (1.35)	2.72 (1.26)	1.775	0.079
19. The dog visits give me energy.	1.86 (1.06)	2.02 (1.18)	0.743	0.459	1.94 (1.12)	1.60 (0.89)	-0.833	0.446	1.94 (1.10)	1.85 (1.21)	-0.267	0.793	1.91 (1.14)	1.98 (1.07)	0.323	0.747
20. I miss the dog visitor between visits.	2.31 (1.42)	2.39 (1.57)	0.281	0.779	2.37 (1.49)	1.80 (1.30)	-0.945	0.393	2.39 (1.49)	2.00 (1.41)	-0.921	0.371	2.27 (1.53)	2.48 (1.40)	0.723	0.472
21. The dog doesn't judge me.	1.23 (0.70)	1.39 (1.06)	0.901	0.371	1.30 (0.89)	1.20 (0.45)	-0.473	0.654	1.27 (0.80)	1.54 (1.33)	0.718	0.485	1.35 (0.94)	1.20 (0.72)	-0.956	0.342
22. I look forward to the dog visits.	1.66 (1.00)	1.84 (1.18)	0.836	0.405	1.72 (1.08)	2.00 (1.22)	0.494	0.646	1.76 (1.10)	1.54 (0.97)	-0.773	0.451	1.73 (1.10)	1.75 (1.06)	0.096	0.923
23. The dog visits make me feel happy.	1.52 (0.77)	1.71 (1.02)	1.096	0.276	1.61 (0.90)	1.40 (0.55)	-0.827	0.446	1.62 (0.90)	1.46 (0.78)	-0.695	0.497	1.59 (0.92)	1.62 (0.84)	0.179	0.859
24. The dog accepts me just the way I am.	1.17 (0.45)	1.39 (0.79)	1.741	0.086	1.26 (0.63)	1.40 (0.55)	0.567	0.598	1.27 (0.63)	1.23 (0.60)	-0.206	0.84	1.30 (0.68)	1.20 (0.52)	-0.858	0.393
25. I make the dog feel better.	2.38 (1.06)	2.31 (1.19)	-0.288	0.774	2.35 (1.12)	2.40 (1.14)	0.104	0.922	2.42 (1.13)	1.75 (0.75)	-2.733	0.014	2.21 (1.16)	2.60 (0.98)	1.892	0.062
26. I make the dog feel happy.	2.23 (1.07)	2.12 (1.14)	-0.516	0.607	2.20 (1.11)	2.00 (0.71)	-0.588	0.582	2.26 (1.11)	1.58 (0.79)	-2.661	0.017	2.01 (1.11)	2.50 (1.01)	2.354	0.021
27. The dog takes my mind off my troubles.	1.97 (1.25)	1.94 (1.42)	-0.121	0.904	1.97 (1.34)	1.60 (0.89)	-0.885	0.418	1.95 (1.30)	2.00 (1.54)	0.108	0.916	1.82 (1.33)	2.20 (1.29)	1.485	0.141
28. The dog helps me feel secure.	2.06 (1.30)	1.92 (1.29)	-0.592	0.555	2.04 (1.30)	1.20 (0.45)	-3.543	0.008	2.03 (1.31)	1.75 (1.14)	-0.792	0.441	1.79 (1.21)	2.38 (1.35)	2.269	0.026

Total score range: from 28 (always true) to 140 (never true). Individual item range: from 1 (always true) to 5 (never true).
